# Ischemic small bowel perforation caused by cholesterol crystal embolism following transcatheter arterial chemoembolization for recurrent hepatocellular carcinoma: a case report

**DOI:** 10.1186/s40792-021-01116-8

**Published:** 2021-02-10

**Authors:** Ryoga Hamura, Koichiro Haruki, Ryota Iwase, Kenei Furukawa, Yoshihiro Shirai, Shinji Onda, Takeshi Gocho, Toru Ikegami

**Affiliations:** Department of Surgery, The Jikei University School of Medicine, 3-25-8, Nishi-Shinbashi, Minato-ku, Tokyo, 105-8461 Japan

**Keywords:** Cholesterol crystal embolism, Transcatheter arterial chemoembolization, Small bowel perforation

## Abstract

**Background:**

Cholesterol crystal embolism (CCE) following transcatheter arterial chemoembolization (TACE) is rare.

**Case presentation:**

A 71-year-old man underwent TACE for recurrence of hepatocellular carcinoma (HCC). On postoperative day (POD) 5, he developed abdominal pain and fever. Computed tomography revealed intraperitoneal free air. The patient was diagnosed with gastrointestinal perforation with peritonitis, for which partial intestinal resection and covering ileostomy were performed. Histological examination revealed perforation of the small intestine caused by CCE. The patient made a satisfactory recovery and was discharged on POD 30. The patient showed no recurrence of cholesterol crystal embolism or HCC for 2 years after surgery.

**Conclusion:**

We reported a successfully treated case of ischemic small bowel perforation due to cholesterol crystal embolism following transcatheter arterial chemoembolization for recurrent HCC.

## Background

Cholesterol crystal embolism (CCE) is an embolic disorder caused by cholesterol crystals detached from atherosclerotic plaques within arteries. The causes of CCE have been reported to include cardiac catheterization, vascular surgery, and use of anticoagulants, but transcatheter arterial chemoembolization (TACE) is a rare trigger. We herein report a successfully treated case of bowel perforation caused by CCE after TACE.

## Case presentation

**Fig. 1:**
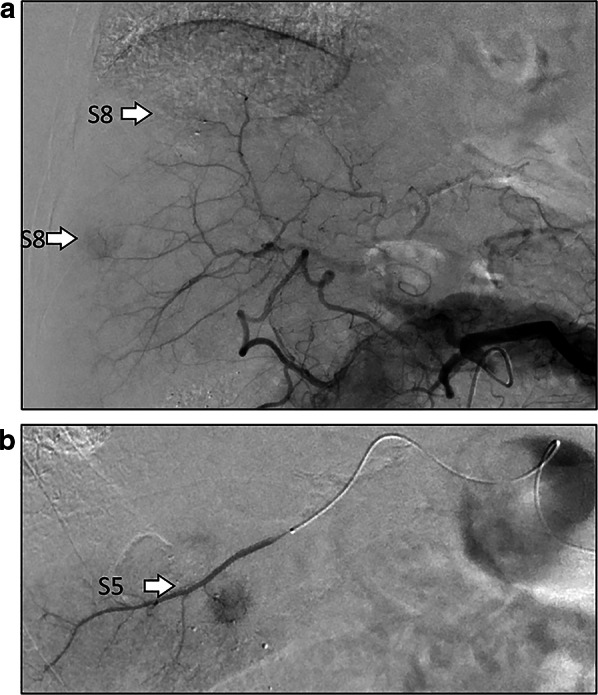
Tumors on S8 (**a**) and S5 (**b**) were embolized through the celiac artery

**Fig. 2:**
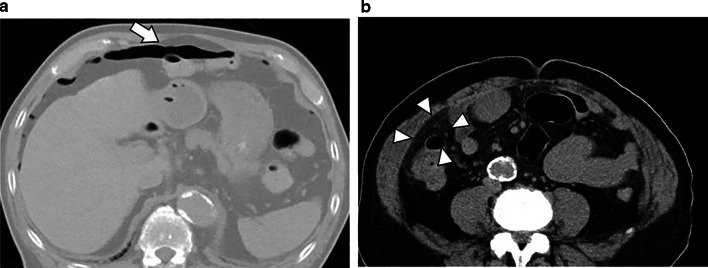
Computed tomography revealed intraperitoneal free air (**a**: arrow) and dirty fat signs around the ascending colon (**b**: arrowhead)

**Fig. 3:**
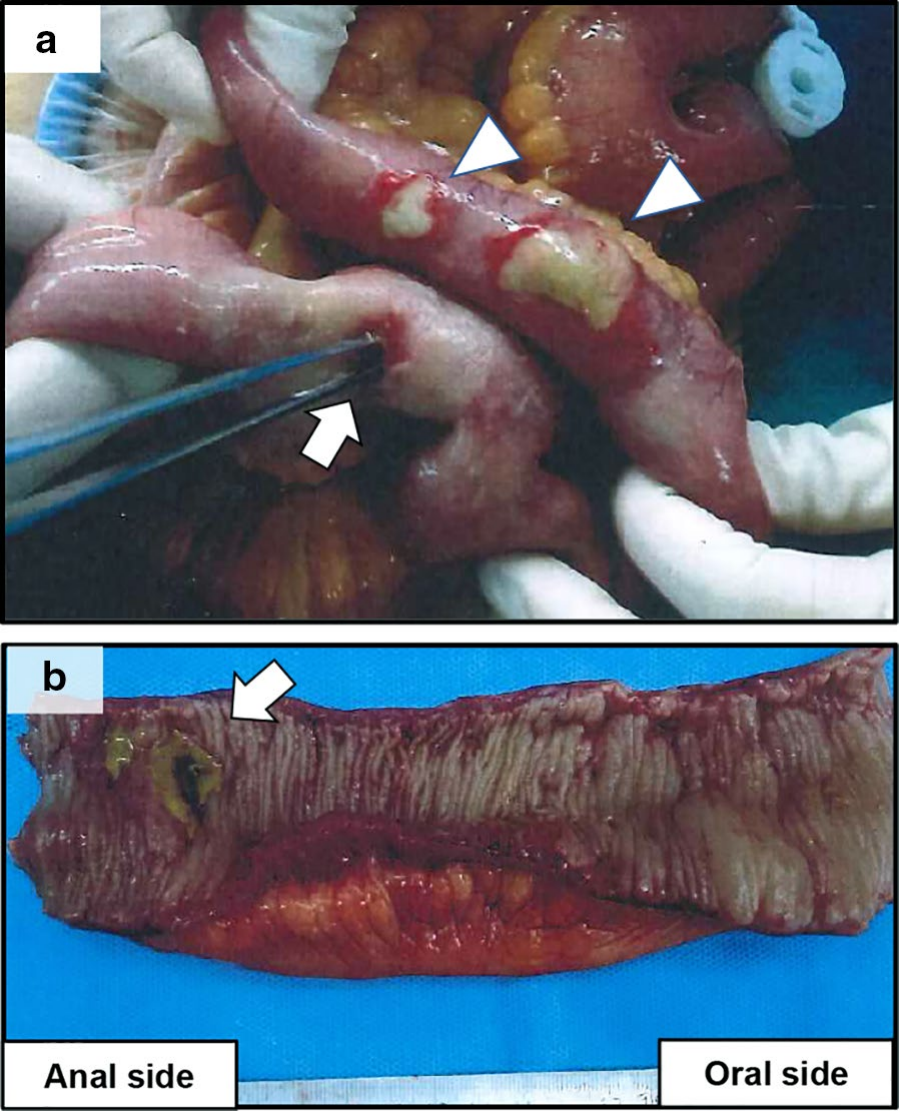
**a** Multiple ulcers (arrowheads) and perforation (arrow) were found on the terminal small bowel. **b** Resected small intestine; perforation in the small intestine (arrow)

**Fig. 4:**
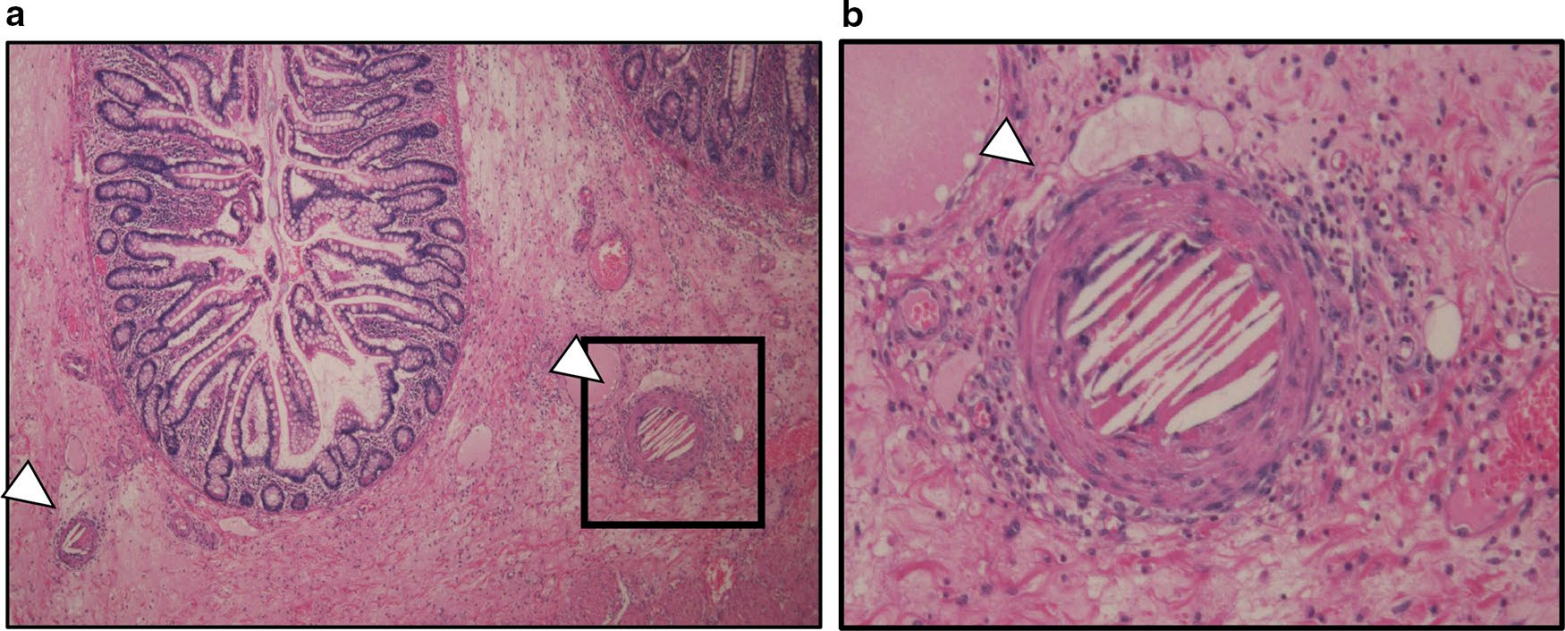
Pathological examination showing cholesterol crystal embolism of the arterial vasculature of the small intestine (arrowheads)

A 71-year-old man on hemodialysis for chronic renal failure due to IgA nephropathy developed recurrent hepatocellular carcinoma (HCC) in segment 5 (S5) and segment 8 (S8) 3 years after hepatic resection followed by TACE. The recurrent HCCs were treated by repeat TACE in which, tumors in S5 and S8 were embolized through the celiac artery (Fig. 1), and the patient was discharged on postoperative day (POD) 4 without any complications. However, on the following day, he was re-admitted to our hospital because of abdominal pain and fever. Laboratory findings showed an increase in white blood cell count to 9.9 × 10^3^/μl and serum C-reactive protein level to 19.91 mg/dl. Abdominal computed tomography (CT) revealed intraperitoneal free air and dirty fat signs around the ascending colon (Fig. 2a, b). The patient was diagnosed with gastrointestinal perforation with peritonitis, and underwent emergency laparotomy. Upon surgical exploration of the abdomen, multiple ulcers and perforation were detected in the terminal ileum (Fig. 3a, b), for which intestinal resection and ileostomy were performed. Pathological examination revealed CCE of arterial vasculature of the small intestine that indicated ischemic perforation of the small intestine caused by CCE (Fig. 4). After surgery, the patient developed superficial surgical site infection caused by methicillin-resistant *Staphylococcus aureus*, which was treated with systemic antibiotics. The patient recovered and was discharged on POD 30. He had no further recurrence of cholesterol crystal embolism or HCC as of 3 years after surgery.

## Discussion

CCE from atherosclerotic plaques is a well-known complication of cardiac catheterization, vascular surgery, and anticoagulation in patients with atherosclerosis and ulcerated aortic plaques (1–5). Risk factors for CCE include male gender, heart failure, peripheral vascular disease, arterial hypertension, diabetes mellitus, and cigarette use (4, 5). The incidence of CCE has been reported 0.18–0.31% during cardiac angiography (2,5), and renal and skin complications caused by micro-arterial vascular embolisms are most frequent during CCE (4–8). Renal failure due to CCE, which occurs several weeks after the cardiovascular procedure, is often progressive and has an extremely poor prognosis (9). Although treatment with anticoagulants, angiotensin 2 antagonists and other vasodilators for hypertension, steroid pulse and/or prostaglandin therapy, and resection of the skin lesion have been considered as treatment options for CCE, no curative treatment for CCE to date has been identified (1–6, 10). Hemodialysis for acute renal failure is required in 40–61% of patients with CCE (3), and aggressive therapies such as hemodialysis have been able to reduce the mortality rate of systemic CCE by as much as 13% within 1 year of diagnosis (3, 11). Gastrointestinal complications are the third most common complication followed by kidney and skin complications (1–5, 12, 13). Common symptoms include abdominal pain, bleeding from mucosal ulcers, and diarrhea. However, it is rare to develop perforation. Furthermore, although surgical resection is the only radical treatment for small bowel perforation due to CCE, postoperative perforation in the remnant small bowel happens frequently (13, 14). The mortality rate of gastrointestinal ischemic ulcer due to CCE is as high as 40% within 1 year, and most patients die of sepsis, shock, and multiple organ system failure (2, 15).

Even if atherosclerotic plaques are scattered in TACE, CCE to the renal, skin, gastrointestinal tract, especially the small intestine is unlikely to occur because TACE is originally a treatment via the celiac artery, and plaque trapped in the liver. In the present case, CCE occurred after TACE and was limited to the terminal ileum. The possible reasons for the CCE to scatter to the terminal ileum is that during catheter intervention in the celiac artery for TACE, an atherosclerotic plaque scattered to the superior mesenteric artery (SMA) via the pancreaticoduodenal arterial arcade, and also the plaque that caused the CCE may have been located in the main trunk of the SMA. Previous report has shown that CCE occurred in the pancreas and duodenum (16), suggesting potential route from celiac artery to SMA trunk through the pancreaticoduodenal arterial arcades. These processes can lead to localized ischemic ulcers and perforation in the small bowel without developing complications such as renal infarction, skin complications, and another intestinal perforation. In this present case, only small bowel perforation was observed by intraoperative exploration. However, if an ischemia or perforation was observed in the ascending or transverse colon, right hemi-colectomy should have been considered.

To the best of our knowledge, this case is the an extremely rare case of CCE following TACE for HCC, which made a satisfactory recovery.

## Conclusion

CCE is a rare but serious complication with poor prognosis caused by catheter intervention in atherosclerosis patients. We reported a successfully treated ischemic small bowel perforation caused by CCE following TACE for recurrent HCC.

## Data Availability

Data sharing is not applicable to this article as no datasets were generated or analyzed during the current study.

## Abbreviations

CCE: Cholesterol crystal embolism
TACE: Transcatheter arterial chemoembolization
HCC: Hepatocellular carcinoma
CT: Computed tomography
POD: Postoperative days
SMA: Superior mesenteric artery
